# The BioChemical Clogging of Landfill Leachate Collection System: Based on Laboratory Studies

**DOI:** 10.3390/ijerph17072299

**Published:** 2020-03-29

**Authors:** Yili Liu, Jianguo Liu

**Affiliations:** 1 School of Automobile, Chang’an University, Xi’an 710064, China; 2 School of Environment, Tsinghua University, Beijing 100084, China

**Keywords:** landfill, leachate collection system (LCS), biochemical clogging, column experiment

## Abstract

Leachate collection system (LCS) clogging is a common operational problem in municipal solid waste (MSW) landfills in China, which can result in high leachate levels that threaten the safety of landfill operations and subsequently increase the leachate leakage risk. In our previous research, a filtration test was conducted and the physical clogging effect was evaluated. To fully analyze the LCS failure, in this study, a set of column experiments were carried out to investigate the biochemical clogging development and mechanisms. Results showed that the biofilm and deposited CaCO_3_ composed the primary clogging materials. During the experimental period, the hydraulic conductivities in simulated gravel and nonwoven geotextile drainage layers were observed (91.7% and five orders of magnitude reduction), and decreased to 10^−4^ and 10^−8^ m s^−1^, respectively. Therefore, the significance of the geotextile layer in LCS designing needs to be reconsidered. The biochemical clogging was positively correlated with volatile fatty acids (VFAs), and Ca^2+^ loading and the Ca^2+^ played the dominant role. Meanwhile, an improved method for analyzing biochemical clogging development was proposed.

## 1. Introduction

In 2018, 1.2 billion tons of municipal solid waste (MSW) was disposed in 663 landfill sites in China. To ensure operational safety and minimize leachate leakage, the leachate collection system (LCS) was designed and installed at the bottom of the modern sanitary landfills. However, field investigations found that most of these landfills are facing leachate drainage challenges and the LCS could become clogged in a short time. The measured leachate output ratio (based on the unit mass of treated waste) was far less than that of the MSW bunkers from the incineration plants. In some of the landfills, the LCS drained much less leachate than expected over several years of operation. A field test showed that the LCS hydraulic conductivity was as low as 10^−8^ m s^−1^ at the Laohukeng sanitary landfill in Shenzhen city [1]. As a result, the leachate level ranged from several to dozens meters in MSW landfills and had caused several landslides events in China [2,3]. Additionally, the saturated waste could block landfill gas (LFG) transport channels and prevent the directional diffusing to the gas collection system [4,5]. Reduced collection efficiency could increase fugitive LFG emissions which would cause a negative impact on not only the local sanitary environment but also on global warming.

According to the formation mechanisms, the LCS clogging could be divided into suspended particulate matter deposition (physical), biofilm growth (biological), and metal ions precipitation (chemical). Because of the causal relationship between the microbial actives and chemical reactions, the last two parts are usually combined and called biochemical clogging. In our previous research, physical clogging was systematically studied by experiments and numerical simulations [6]. However, the other biochemical clogging mechanism was yet to be studied.

The existing studies showed that the biochemical clogging development was remarkable in both the geotextile [7] and gravel [8] layers. Through leachate-permeated experiments, Palmeira et al. reported that the hydraulic conductivity of three types of nonwoven geotextile could decrease by three to four orders of magnitude within one hundred days [9], and Rowe et al. found that the excessive microbially induced clogging near the influent end of the simulated gravel column, resulting in a decrease in hydraulic conductivity by seven orders of magnitude [10].

The biochemical clogging mainly included biofilm formation and CaCO_3_ precipitation [11], which were closely linked to a series reaction of microbial degradation and chemical precipitation [12,13]. Vangulck and Rowe discovered that there was a significant positive correlation between the precipitation of metal ions and the degradation of volatile fatty acids (VFAs) in leachate [14]. Based on that, a fixed coefficient was suggested to describe the relationship between the Ca^2+^ deposition and the VFAs’ removal or H_2_CO_3_ generation [15]. Unfortunately, this vital parameter was easily affected by many variables such as the Ca^2+^ and TIC (total inorganic carbon) concentrations in the leachate as well as the TIC existent form determined by the pH value. Therefore, even under the same experimental conditions, the numerical deviation of this ratio was more than one order of magnitude [16], which limited further application in the numerical model development.

Meanwhile, the higher food waste proportion in Chinese MSW was responsible for more leachate generation and higher VFAs concentration [17,18], which accelerated the biochemical clogging. Therefore, in this study, a set of simulated column experiments was carried out to analyze the biochemical clogging effect on the Chinese LCS and to investigate the clogging mechanisms. Furthermore, an improved calculation method was proposed to be the basis of subsequent numerical simulation.

## 2. Materials and Methods

### 2.1. Grouping and Operation

The typical LCS consists of the geotextile layer, gravel layer, and drainpipe from top to bottom (Figure S1). Based on the LCS designing criteria and leachate characteristics, four groups of experimental columns were set up and there were two parallel columns in each group. Among them, the gravel (G) and nonwoven geotextile (N) groups were aimed to study biochemical clogging development. Compared to group G and N, by reducing the leachate flow rate or improving the Ca^2+^ concentration in the synthetic leachate artificially, the “lower load” group (L) and the “higher calcium concentration” group (H) were used for analyzing the influencing factors.

**Figure 1:**
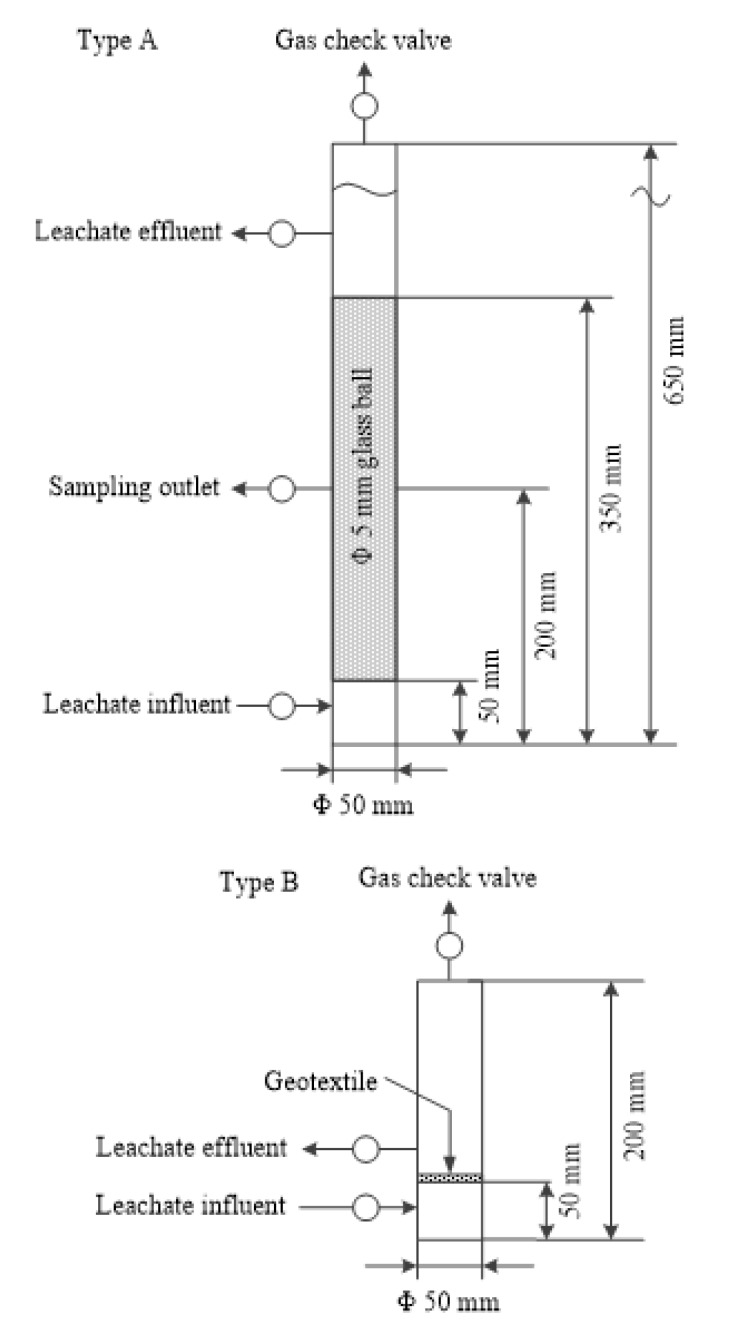
The schematic diagram of the simulation columns of leachate collection system (LCS).

Two types of columns were designed as follows (Figure 1). Type A, for group G, L, and H, was filled with 5 mm glass balls to simulate the gravel drainage layer. Type B, for group N, was loaded with 400 g m^−2^ nonwoven geotextile, which was commonly used in Chinese landfills, and the detailed parameters of the geotextile were initial hydraulic conductivity 10^−3^ m s^−1^, initial porosity 0.9, and equivalent aperture 0.05–0.20 mm. The diameter of these columns was 50 mm, and a gas check valve was installed at the top of each column as the “biogas” outlet to maintain anaerobic conditions.

Before the experiment, these columns were filled with real leachate from the Xiaowuji MSW transfer station for two weeks for microbial inoculation. During the experiment, the synthetic leachate (without particulate matter) was pumped into each column from the leachate influent end and collected at the leachate effluent end. The average hydraulic load of these columns was 0.51 m^3^ m^−2^ d^−1^ for G, N, and H groups [19] and 0.255 m^3^ m^−2^ d^−1^ for L group. This experiment was operated continuously under dark conditions (avoiding algae growth) and at room temperature (15–35 °C) for 220 days until the hydraulic conductivity of the geotextile became lower than 10^−8^ m s^−1^ (Figure S2).

According to the primary leachate test results, the synthetic leachate for groups G, N, and L were prepared by analytically pure chemicals. The NaHCO_3_ and Na_2_CO_3_ were used to adjust the pH value and replenish the TIC in the liquid phase to fully simulate the characteristics of real landfill leachate. The microelements were added following the recommended values outlined by Vangulck and Rowe [19]. The pH value, as well as the concentrations of VFAs (i.e., acetate, propionate, and butyrate), Ca^2+^, Mg^2+^, and TIC of the influent leachate, were measured periodically and listed in Table 1.

Furthermore, the CaCl_2_ solution was added to the influent leachate of group H; the measurement results are shown in Table 2.

### 2.2. Testing Methods

During the experiment, the hydraulic conductivity and the chemical compositions of sampled leachate from the influent, middle, and effluent of each column were tested regularly to study the clogging development in the gravel and nonwoven geotextile layers. At the end of the experiment, the precipitate properties were detected to analyze the clogging mechanism.

When testing the hydraulic conductivity, two methods of falling head and constant head were duly adopted according to the filtration velocity [20]. For the liquid samples, the pH value was measured by a Fe20 pH electrode (Mettle, Switzerland); the total organic carbon (TOC) and TIC were tested using a TOC-VCPH instrument (Shimadzu, Japan); the VFAs were obtained by gas chromatography (GC, Shimadzu, Japan) with flame ionization detectors, and the Ca^2+^ and Mg^2+^ concentrations were analyzed by inductively coupled plasma-atomic emission spectrometry (ICP-AES, Perkin Elmer, USA).

For the solid samples, the ratio of organic to inorganic substances was measured by weighing after being dried at 105 and 550 °C; the elemental composition and crystal structure of the inorganic part were studied by X-ray fluorescence spectrometer (XRF, Thermo Fisher, USA) and X-ray diffraction (XRD, PAN alytical X’Pert, The Netherlands), respectively.

## 3. Results and Discussion

### 3.1. Clogging Development

**Figure 2:**
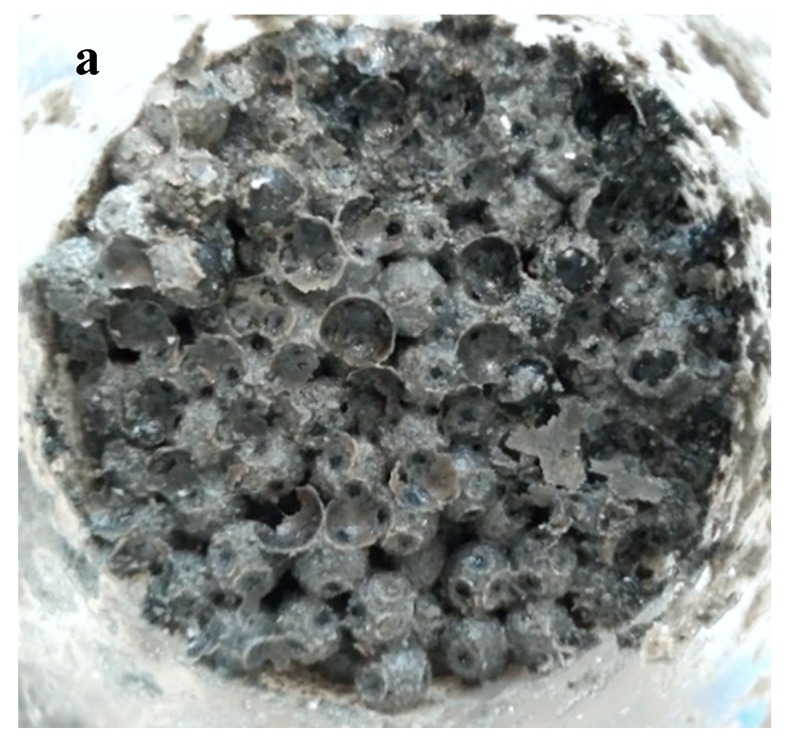

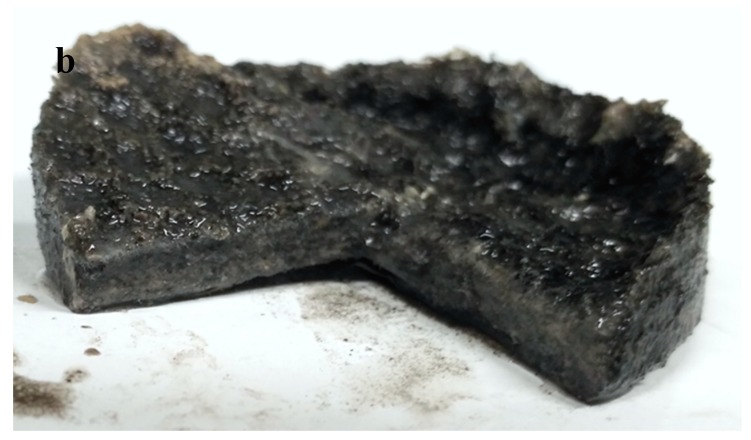
Photos of the clogging materials: (**a**) simulated gravel layer and (**b**) nonwoven geotextile layer.

**Figure 3:**
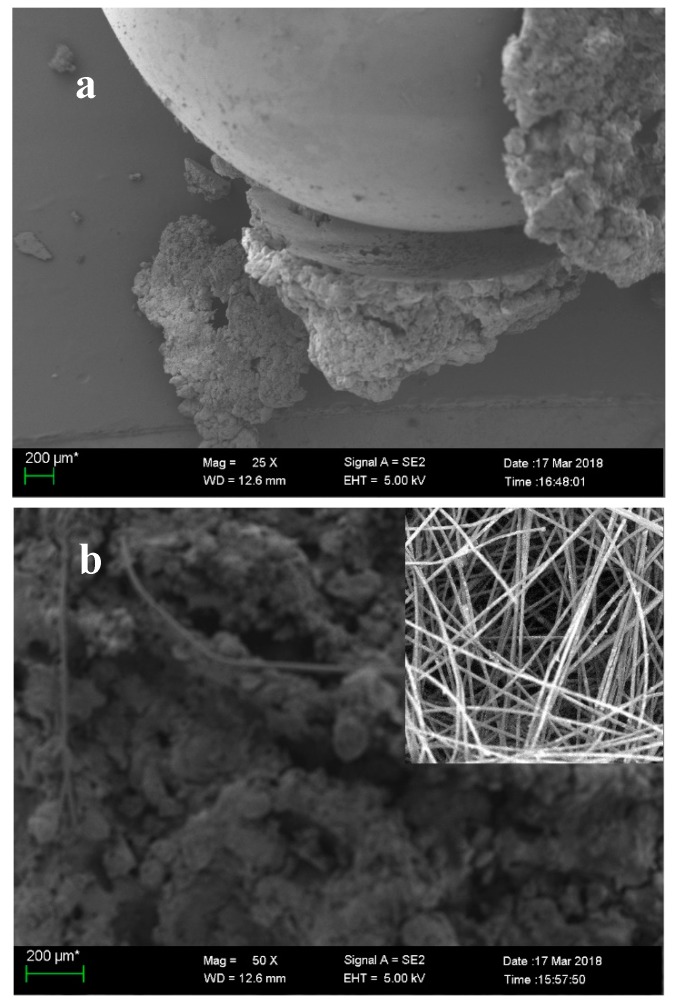
SEM pictures of the clogging materials: (**a**) the surface of glass spheres and (**b**) nonwoven geotextile fibers (upper-right corner is the SEM pictures of nonwoven geotextile before the experiment).

The photos and scanning electron microscopy (SEM) pictures directly showed the biochemical clogging of the drainage mediums and the precipitates form. In the simulated gravel layer, the surface of glass spheres was enveloped by the precipitates regularly, which further interconnected with one another and blocked the drainage channels (Figure 2a and Figure 3a). In the nonwoven geotextile layer, the deposited biofilms and inorganic substances coated the geotextile fibers layer by layer and filled up the internal pore eventually (Figure 2b and Figure 3b). Meanwhile, the profile photos of the geotextile exhibited the uniform distribution of the precipitates.

**Figure 4:**
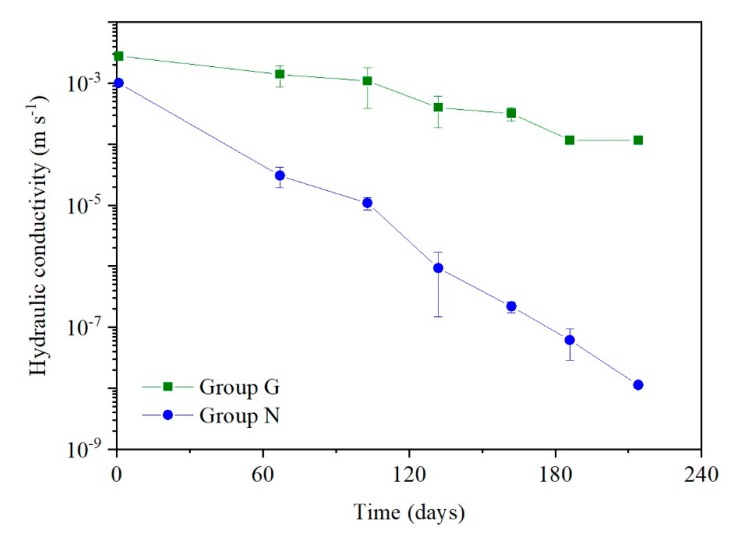
The hydraulic conductivity variation of the groups G and N.

The hydraulic conductivity changes in group G and N during the continuous experiment over 220 days are shown in Figure 4.

The biochemical clogging developed at a slower rate in the gravel drainage layer (group G), and the total permeability loss of the 30 cm thickness glass bed was 91.7%. However, another approximate experiment reported that the hydraulic conductivity of 4, 6, and 15 mm glass spheres could decrease by 7–8 orders of magnitude during 250 days [21]. Based on the follow-up study results, the difference between these two experiments can be explained as follows: (1) the real landfill leachate was irrigated into the columns in that experiment, which made the clogging mechanism hard to distinguish; (2) caused by the microbial growth differences, the biofilm content was only 36.5% of Rowe‘s experiment and the biological clogging was eased (Figure S3); (3) along with less biofilm content, the COD (TOC) degradation rate was reduced to about one fourth and the lower TIC generation amount decelerated the chemical precipitation; (4) because of the lower pH value of the synthetic leachate, limited CO_3_^2−^ was ionized from the generated TIC and the chemical clogging was restricted; (5) the experimental observations difference was magnified by the exponential correlation between the hydraulic conductivity and residual porosity [14].

Since the nonwoven geotextile takes up a larger specific surface area, it could be a suitable carrier for microbial growth. At the end of the experiment, the percentage of dried organic sediments (biological clogging) was 15.8–17.4%, which was about three times larger than in the simulative gravel layer. Even though the moisture content (wet base) was tested at 50.7–55.1%, the majority of the water was combined with the biofilm (intracellular water) and was difficult to drain. Biofilm was not the only possible source to fill the drainage pores but also the CO_3_^2−^ generated by the biofilm romoted the further precipitation with Ca^2+^ to form CaCO_3_, another main constituent in the precipitates. Over the experiment period, the hydraulic conductivity of the nonwoven geotextile decreased with time (leachate flux) at an equal-ratio to 10^−8^ m s^−1^ after 220 days (Group N). As a result, even the physical clogging was excluded; the geotextile also degenerated into the water-permeable layer under the biochemical effect and enabled the leachate accumulation within the landfill body.

Whether the LCS designing criteria [22,23,24] fit for the landfills with higher leachate yield and organic concentrations, such as the landfills in developing countries or bioreactor landfills, should be reconsidered. Although the geotextile could extend the service life of the gravel layer by preventing particle invasion, it would become an aquiclude in a short time under the combined factors of biochemical clogging as well as physical clogging [6] and the mechanical load of landfilled waste layers [25]. Thus, the main problem of “water management” in the early stage of landfills was to drain more leachate into the LCS and reduce the saturation in the landfill body rather than to lower the leachate head on the bottom liner. As a result, the role of geotextile had to be balanced.

Besides, this biochemical clogging experiment was carried out under room temperature (15–35 °C). However, the reported temperature values ranged from 30 to 50 °C for MSW landfills [26] and 30 to 60 °C [27] for bioreactor landfills. The higher temperature not only improved the biological activity of microorganisms [28] but also reduced the solubility of CaCO_3_ [29]. Thus, the biochemical clogging development in real landfills might be more rapid than the experiment result.

### 3.2. Influence Factors

**Figure 5:**
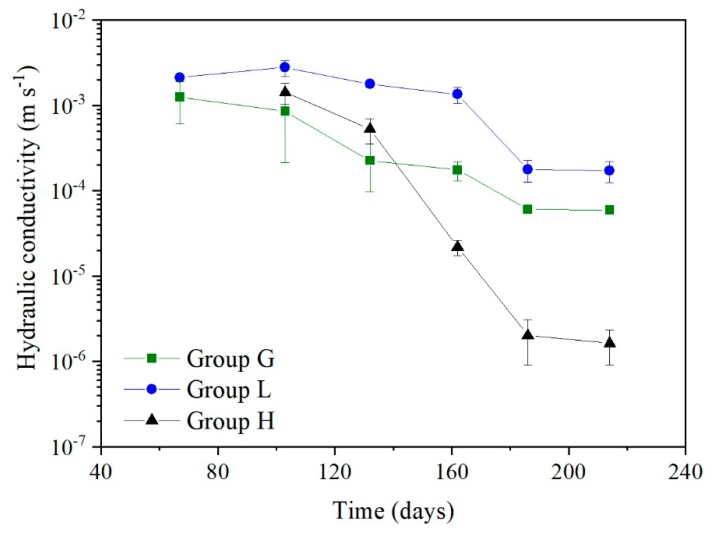
The hydraulic conductivity variation of the groups G, L, and H.

The declining tendency of hydraulic conductivity in groups G, L, and H showed that (1) by reducing the hydraulic (mass) load to half, the residual permeability of group L roughly doubled than group G and (2) when the concentration of VFAs decreased by 49.5% and Ca^2+^ increased by 280.1%, the hydraulic conductivity in group H was only 2.3% of that in group G (Figure 5). These results indicated that both the organic matter and Ca^2+^ loads had significant effects on the biochemical clogging development. Nevertheless, the Ca^2+^ played the dominant role.

**Figure 6:**
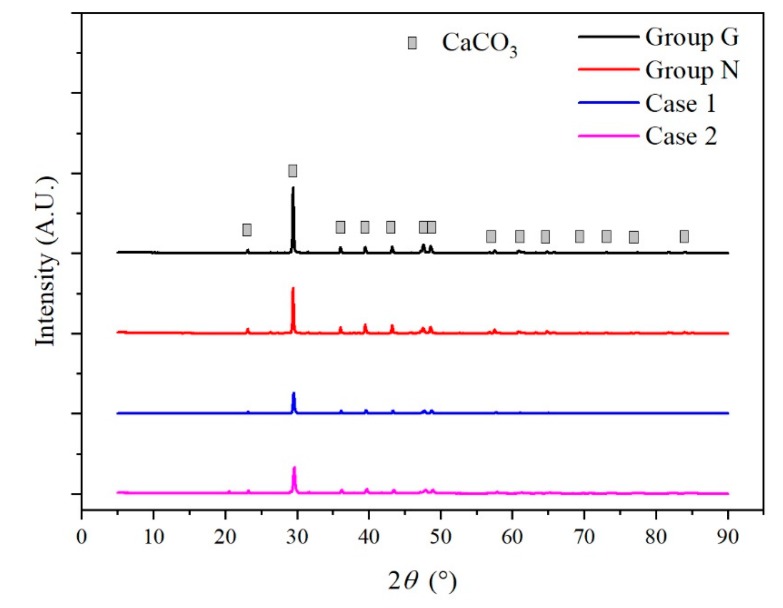
Crystal structure of the inorganic precipitates.

The XRF detection results presented that the inorganic precipitate mainly consisted of Ca and C, and these two elements had a molar ratio of nearly 1:1 (Table S1). By comparing the main peaks in different diffraction angles (2*θ*), the XRD analysis confirmed that the CaCO_3_ was the main crystal structure (Figure 6).

Due to the solubility product limitation, the precipitation of Mg^2+^ was weak and had little effect on the biochemical clogging. Besides, the detection results of the sediments sampled, respectively, from the leachate drainpipes of different cities (Shanghai and Wuhan) showed good agreement with the column experiment results (Table S1). 

Compared with developed countries, the higher proportion of food waste (Table S2) in China results in the generation of more leachate per ton of waste and leads to a higher concentration of VFAs, which accelerates biochemical clogging. MSW classified collection and avoidance primary food waste disposal could help prolong LCS service life.

Moreover, with the rapid growth of sewage treatment rate and waste incineration ratio, a large number of lime-dried dewatered sludge and incinerated ash residue are disposed of and cause two negative effects on the LCS. Firstly, the Ca^2+^ concentration in the leachate increase and the Ca^2+^ limitation is be released in clogging development. Secondly, these high calcium-containing wastes are usually alkaline, which promotes the ionization of H_2_CO_3_ and HCO_3_^−^ into CO_3_^2−^ and the generation of CaCO_3_ by improving the leachate pH value. These kinds of waste should be isolated from the MSW landfill and equipped with an independent leachate drainage system.

### 3.3. Clogging Processes

**Figure 7:**
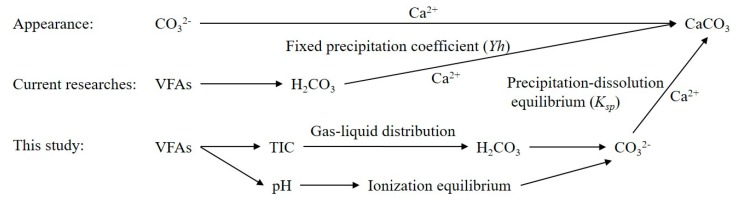
The calculating paths of biochemical reactions.

Both the literature and column experiment results indicated that the precipitated CaCO_3_ was the most important inorganic component in the biochemical clogging. Vangulck et al. developed a linear regression of the measured removal of Ca^2+^ versus the calculated H_2_CO_3_ production (TIC generation in this study) and suggested that the yield coefficient (*Y_H_*) reflected this relationship [16]. In the following studies, this fixed coefficient (*Y_H_*) was adopted in the development of clogging predicting models [15,30]. However, the reaction path from biodegradation to chemical precipitation was concatenated and complicated. The single proportion factor *Y_H_* only represented the apparent result of the multiple reactions and effects at a certain condition. When the reaction conditions changed, this parameter was affected significantly. As a result, even under the same experimental conditions, the numerical deviation was more than one order of magnitude [16]. For this reason, this ratio was difficult to ascertain, which limited these model applications. Hence, the causal link between the VFAs degradation and Ca^2+^ precipitation was further studied and another calculating path of biochemical reactions was carried out along the reactions sequence as follows (Figure 7).

#### 3.3.1. TIC Generation and Retention

In liquid, the VFAs (acetate, propionate, and butyrate) constitute the majority of the degradable TOC and are the main source of TIC. According to the fermentation path, 1mol acetate, propionate, and butyrate release 1, 1.25, and 1.5 mol TIC in anaerobic degradation [31,32]. Next, considering the gas–liquid distribution equilibrium, only parts of the generated TIC migrate with the leachate or generate carbonate. In reality, this equilibrium is easily and greatly influenced by many factors, such as temperature, gases partial pressure, water saturation, and reactions in the liquid. 

Here, a retention coefficient, *ψ_AL_*, was defined to represent the retention percentage of the generated TIC in the leachate or precipitates. In this study, the TIC that remained or used to remain in the liquid was calculated by the sum of the measured TIC concentration change and the absolute value of Ca^2+^ removal (calculated by molar concentration and assuming all the inorganic carbon conserved in CaCO_3_ was from the dissolved fraction) among different sampling points. The total generated TIC was calculated by the measured VFAs loss. After this, the ratio between them was *ψ_AL_*.

**Figure 8:**
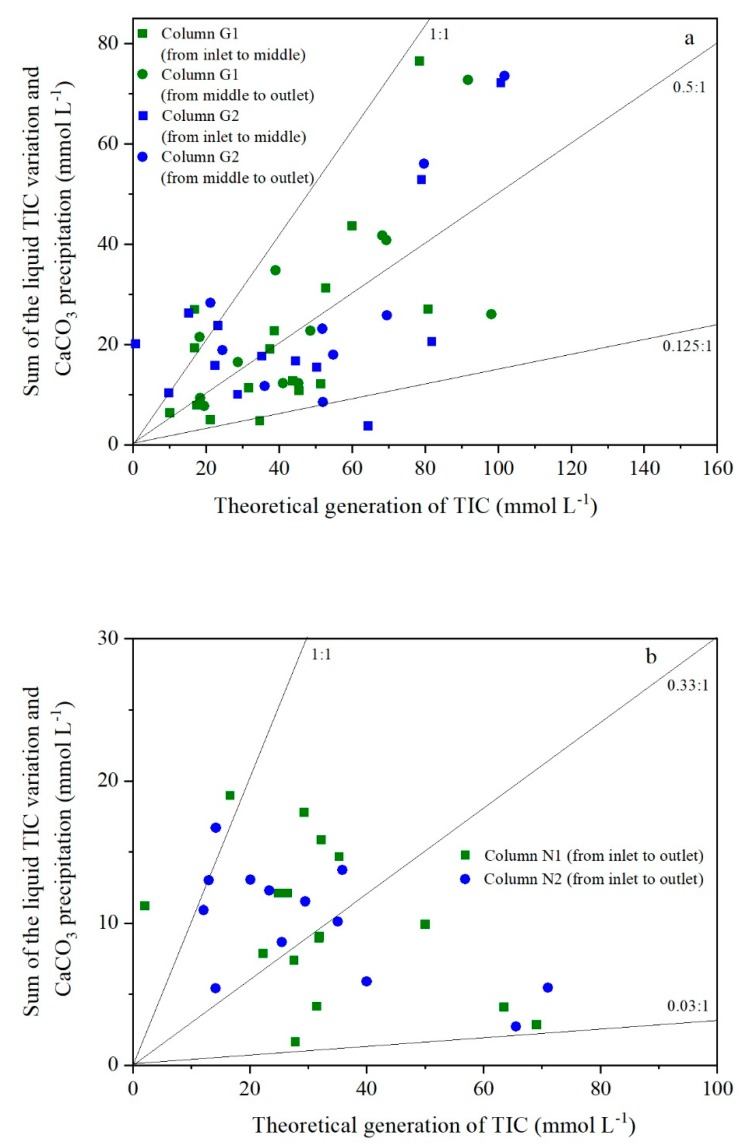
Total inorganic carbon (TIC) retention ratio: (**a**) group G and (**b**) group N.

In Figure 8, this value is roughly between 0.125 and 1 and the mean value was about 0.5 for group G. However, because the water level above the geotextile was limited and made the generated TIC more easily to release into the air phase, the *ψ_AL_* value fluctuated and was relatively smaller for group N. Therefore, the scattered distribution of this parameter might be the cause of the irregular proportion coefficient (*Y_H_*) between the TIC generation and Ca^2+^ removal. Since the value of *ψ_AL_* is affected by many factors and difficult to be quantified by anyone formula, a realistic value could be estimated by the following method:

(1) The volume fraction of CH_4_ and CO_2_ of the LFG is usually considered to be 55% and 45%, respectively [33,34,35].
(2) The measured volume ratio of CH_4_ and CO_2_ of the LFG in China is about 1.75–2 [36,37].
(3) The difference between 1) and 2) is considered that parts of the generated TIC are still dissolved in the liquid phase and are discharged with the leachate due to the higher water saturation in Chinese landfills. The value of *ψ_AL_* is about 30.1–38.9% by mathematical calculation.

#### 3.3.2. pH Value Changes

**Figure 9:**
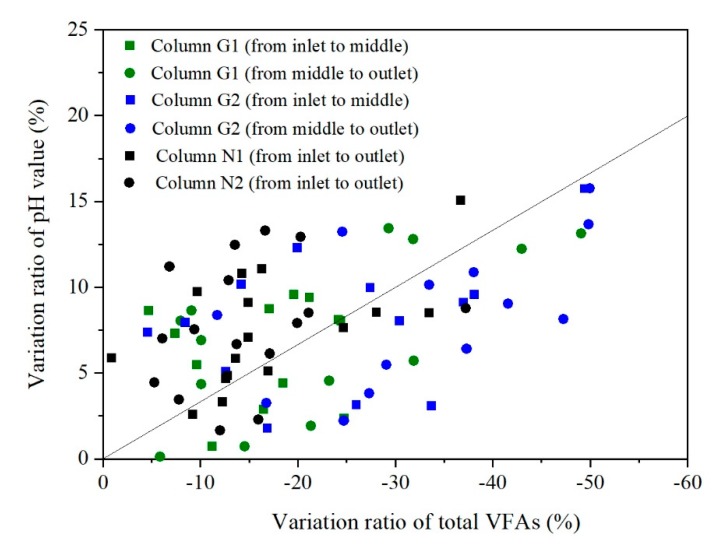
The relationship between pH value and total volatile fatty acids (VFAs) variation.

Because the acidity of carbonate is significantly lower than that of acetic acid, propionic acid, and butyric acid, the pH value of the leachate gradually rose with the VFAs degradation. When the pH value changed from 6.5 to 7.5, the ionized CO_3_^2−^ ratio increased by an order of magnitude, which further caused an important impact on the biochemical clogging reactions. In this study, the maximum pH value variation was measured about 1.3 from the outlet to inlet, and a significant negative correlation between the changes of pH value and total VFAs concentrations was observed (Figure 9). According to the column experiment results, a calculation formula was proposed to reflect this relationship:

(1) $$pH=pH_{0}\times \left(1+\psi_{pH}\frac{\sum_{i=1}^{3}\Delta VFA_{i}}{\sum_{i=1}^{3}VFA_{0i}}\right)$$

Here, *pH*_0_ (no unit) and *VFA*_0_ (mmol L^−1^) are the initial pH and VFA concentration of the inflow leachate. ∆*VFA* (mmol L^−1^) is the VFA concentration variation during the leachate drainage. The *i*, from 1 to 3, represents acetic acid, propionic acid, and butyric acid, respectively. The coefficient, *ψ_pH_* (no unit), indicates the corresponding change between the pH value and the total VFAs concentrations and the number was suggested as minus one third.

#### 3.3.3. CaCO_3_ Precipitation

**Figure 10:**
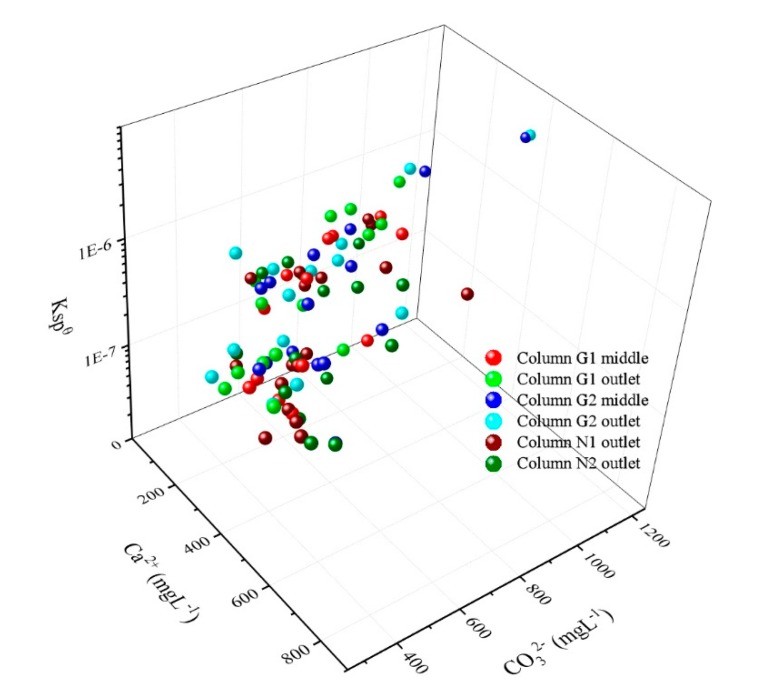
The measured critical solubility product of CaCO3 precipitation.

When the CO_3_^2−^ concentration gradually increased with biological reactions and the associated product and Ca^2+^ concentration went beyond a certain value (*K_sp_*), the dynamic equilibrium of the two materials concentrations in the liquid was maintained by producing CaCO_3_ precipitation. Theoretically, the solubility product constant of CaCO_3_ was about 4.9 × 10^−9^ (T = 25 °C) [29]. In reality, a supersaturated condition is required to precipitate and the supersaturation value is extremely high for most compounds. Therefore, the apparent solubility product constant is usually 10^2^–10^3^ higher than the theoretical value [38]. Based on the measured Ca^2+^, TIC concentration, and pH value in each sampling point at each time, the CaCO_3_ precipitation still conformed to the solubility product constant and the critical *K_sp_* was calculated to be 4.3 ± 2.0 × 10^−7^ within the wide range of concentrations (Figure 10). Meanwhile, this simulated experimental result was consistent with the testing value (5.09 ± 4.77 × 10^−7^) of the leachate samples from 11 landfill sites located in different regions of China.

## 4. Conclusions

In this study, a set of column experiments were carried out to investigate the biochemical clogging development and mechanisms. The results showed that the biofilm and deposited CaCO_3_ composed the primary clogging materials. Because the nonwoven geotextile was a suitable carrier for microbial growth, the hydraulic conductivity of it decreased by five orders of magnitude during the experimental period. Correspondingly, only one order of magnitude reduction of hydraulic conductivity was observed in the simulated gravel layer. The significance of the geotextile layer in LCS needs to be reconsidered. 

The biochemical clogging was positively correlated to VFAs and Ca^2+^ loading. Determined by the leachate characteristics of higher VFAs and lower Ca^2+^ concentration in China, the Ca^2+^ played the dominant role in CaCO_3_ generation. Therefore, the high calcium-containing wastes such as lime-dried dewatered sludge and incinerated ash residue should be isolated from the MSW landfill and equipped with an independent leachate drainage system.

Moreover, in this study, an improved calculation path of biochemical clogging, involving TIC retention, pH value changes with total VFAs, and solubility product of CaCO_3_, was proposed and the key parameters were studied, which could be the foundation of the following numerical models.

## Figures and Tables

**Table 1:** The properties of the synthetic leachate for groups G, N, and L (mg L^−1^, except pH value).

	pH	TIC	TOC	Ca^2+^	Mg^2+^	Acetate	Propionate	Butyrate
Average	6.9	378.0	6176.7	616.3	121.7	5182.6	2337.1	4634.5
Standard deviation	0.28	282.0	1532.6	209.4	16.5	459.8	286.9	377.8

**Table 2:** The properties of the synthetic leachate for group H (mg L^−1^, except pH value).

	pH	TIC	TOC	Ca^2+^	Mg^2+^	Acetate	Propionate	Butyrate
Average	7.0	230.3	3121.5	1726.5	74.0	2611.3	1182.9	2353.8
Standard deviation	0.16	165.0	766.6	490.3	22.1	242.1	145.0	164.6

## Supplementary Materials

The following are available online at https://www.mdpi.com/1660-4601/17/7/2299/s1, Figure S1: Cross-sectional diagram of the leachate collection system, Figure S2. Photo of experimental columns operation, Figure S3. The organic–inorganic sediments content in different parts of experimental columns, Table S1. Elements mass percentage of the inorganic precipitates, Table S2. Food waste proportion in different countries.

## References

[1] Sui J., Huang S., Fang Z., Lin J., Zhu G. (2013). Analysis and case study on sanitation landfill clogging problem based on leachate flow rate variation. Water Wastewater Eng..

[2] Lan J. (January 2012). Mechanism of Leachate Generation, Transport and Mound in MSW Landfills and Control of Leachate Level. Ph.D. Thesis.

[3] Peng R., Hou Y., Zhan L., Yao Y. (2016). Back-Analyses of Landfill Instability Induced by High Water Level: Case Study of Shenzhen Landfill. Int. J. Environ. Res. Public Health.

[4] El-Fadel M., Findikakis A.N., Leckie J.O. (1997). Gas simulation models for solid waste landfills. Crit. Rev. Environ. Sci. Technol..

[5] Zhan T., Xu X., Chen Y., Ma X., Lan J. (2015). Dependence of gas collection efficiency on leachate level at wet municipal solid waste landfills and its improvement methods in China. J. Geotech. Geoenviron. Eng..

[6] Liu Y., Sun W., Du B., Liu J. (2018). The physical clogging of the landfill leachate collection system in China: based on filtration test and numerical modelling. Int. J. Environ. Res. Public Health.

[7] Vieira J., Abramento M., Campos M. Experimental study of clogging in drainage systems. Proceedings of the 9th International Conference on Geosynthetics.

[8] Fleming I., Rowe K. (2004). Laboratory studies of clogging of landfill leachate collection in drainage systems. Can. Geotech. J..

[9] Palmeira E.M., Remigio A.F.N., Ramos M.L.G., Bernardes R.S. (2008). A study on biological clogging of nonwoven geotextiles under leachate flow. Geotext. Geomembr..

[10] Rowe R.K., Vangulck J.F., Millward S.C. (2002). Biologically induced clogging of a granular medium permeated with synthetic leachate. J. Environ. Eng. Sci..

[11] Wu H., Wang Q., Ko J.H., Xu Q. (2018). Characteristics of geotextile clogging in MSW landfills co-disposed with MSWI bottom ash. Waste Manag..

[12] Ko J.H., Wang Q., Yuan T., Wu H., Xu Q. (2019). Geotextile clogging at different stages of municipal solid waste landfills co-disposed with bottom ash. Sci. Total. Environ..

[13] Li Z. (2014). Modeling precipitate-dominant clogging for landfill leachate with NICA-Donnan theory. J. Hazard. Mater..

[14] VanGulck J.F., Rowe R.K. (2004). Influence of landfill leachate suspended solids on clog (biorock) formation. Waste Manag..

[15] Cooke A.J., Rowe R.K., Rittmann B.E. (2005). Modelling species fate and porous media effects for landfill leachate flow. Can. Geotech. J..

[16] Van Gulck J.F., Rowe R.K., Rittmann B.E., Cooke A.J. (2003). Predicting biogeochemical calcium precipitation in landfill leachate collection systems. Biodegradation.

[17] Xing W., Lu W., Zhao Y., Zhang X., Deng W., Christensen T. (2013). Environmental impact assessment of leachate recirculation in landfill of municipal solid waste by comparing with evaporation and discharge (EASEWASTE). Waste Manag..

[18] Xu S., Liu J. (2014). Research on early leachate generation characteristics using deuterium stable isotope tracer technique. Adv. Mater. Res..

[19] VanGulck J.F., Rowe R.K. (2004). Evolution of clog formation with time in columns permeated with synthetic landfill leachate. J. Contam. Hydrol..

[20] Pedescoll A., Samso R., Romero E., Puigagut J., García J. (2011). Reliability, repeatability and accuracy of the falling head method for hydraulic conductivity measurements under laboratory conditions. Ecol. Eng..

[21] Rowe K., Armstrong M., Cullimore D. (2000). Particle size and clogging of granular media permeated with leachate. Am. Soc. Civ. Eng..

[22] United States Environmental Protection Agency (EPA) Avoiding Failure of Leachate Collection and Cap Drainage Systems. https://cfpub.epa.gov/si/si_public_record_Report.cfm?Lab=ORD&dirEntryId=41613.

[23] Environmental Protection Agency (EPA) Criteria for Municipal Solid Waste Landfills. 40CFR258. https://www.ecfr.gov/cgi-bin/text-idx?tpl=/ecfrbrowse/Title40/40cfr258_main_02.tpl.

[24] United States Environmental Protection Agency (EPA) Requirements for Hazardous Waste Landfill Design, Construction, and Closure. https://cfpub.epa.gov/si/si_public_record_report.cfm?Lab=NRMRL&dirEntryId=40449.

[25] Koda E., Miszkowska A., Stępień S. Quality control of non-woven geotextiles used in a drainage system in an old remedial landfill. Proceedings of the Geo-Chicago, Sustainability and Resiliency in Geotechnical Engineering.

[26] Southen J., Rowe K. (2005). Modelling of thermally induced desiccation of geosynthetic clay liners. Geotext. Geomembr..

[27] Yazdani R., Kieffer J., Akau H. (2002). Full Scale Landfill Bioreactor Project at the Yolo County Central Landfill.

[28] Bian X., Liu J. (2014). Influence factors in clogging of landfill leachate collection system. Adv. Mater. Res..

[29] Yuan W., Chi Y., Xin J. (2001). Inorganic Chemistry.

[30] Rowe R.K., Yu Y. (2013). Modeling of leachate collection systems with filter separators in municipal solid waste landfills. J. Environ. Eng..

[31] Parkin G.F., Owen W.F. (1986). Fundamentals of anaerobic digestion of wastewater sludges. J. Environ. Eng..

[32] Bjerg P.L., Ruegge K., Pedersen J.K., Christensen T.H. (1995). Distribution of redox-sensitive groundwater quality parameters downgradient of a landfill (Grindsted, Denmark). Environ. Sci. Technol..

[33] Amini H.R., Reinhart D.R., Mackie K.R. (2012). Determination of first-order landfill gas modeling parameters and uncertainties. Waste Manag..

[34] Amini H.R., Reinhart D.R., Niskanen A. (2013). Comparison of first-order-decay modeled and actual field measured municipal solid waste landfill methane data. Waste Manag..

[35] Krautwurst S., Gerilowski K., Jonsson H., Thompson D., Kolyer R., Iraci L., Thorpe A., Horstjann M., Eastwood M., Leifer I. (2017). Methane emissions from a Californian landfill, determined from airborne remote sensing and in situ measurements. Atmos. Meas. Tech..

[36] Chai X., Zhao X., Lou Z., Takayuki S., Hirofumi N., Cao X., Zha Y. (2011). Characteristics of vegetation and its relationship with landfill gas in closed landfill. Biomass Bioenerg..

[37] Yang L., Chen Z., Zhang X., Liu Y., Xie Y. (2015). Comparison study of landfill gas emissions from subtropical landfill with various phases: A case study in Wuhan, China. J. Air. Waste Manag. Assoc..

[38] Drela I., Falewicz P., Kuczkowska S. (1998). New rapid test for evaluation of scale inhibitors. Water Res..

